# Chemical composition of essential oils of eight Tunisian *Eucalyptus* species and their antibacterial activity against strains responsible for otitis

**DOI:** 10.1186/s12906-021-03379-y

**Published:** 2021-08-12

**Authors:** Elaissi Ameur, Moumni Sarra, Derbali Yosra, Kouja Mariem, Abid Nabil, Frederic Lynen, Khouja Mohamed Larbi

**Affiliations:** 1 Chemical, Pharmacological and Gallenic Development Laboratory, University of Monastir, Faculty of Pharmacy, Avenue Avicennne, 5019 Monastir, Tunisia; 2 University of Carthage, The National Research Institute of Rural Engineering, Water and Forestry, INRGREF, Laboratory of Management and Valorization of Forest Resources, BP 10, 2080 Ariana, Tunisia; 3 Laboratory of Transmissible Diseases and Biological Active Substances LR99ES27, Faculty of Pharmacy, University of Monastir, Monastir, Tunisia; 4 High Institute of Biotechnology of Sidi Thabet, University of Manouba, Manouba, Tunisia; 5 Separation Science Group, Department of Organic and Macromolecular Chemistry, Faculty of Sciences, Ghent University, Krijgslaan 281-S4 Bis, B-9000 Ghent, Belgium

**Keywords:** *Eucalyptus*, Essential oils, Chemical composition, Antibacterial activity, Otitis, Principal Component Analysis (PCA), Hierarchical Cluster Analysis (HCA)

## Abstract

**Background:**

The chemical composition and biological activity of Eucalyptus essential oils have been studied extensively (EOs). A few of them were tested for antibacterial effectiveness against otitis strains. The chemical composition and antibacterial activity of the EOs of eight Tunisian *Eucalyptus* species were assessed in the present study.

**Methods:**

Hydrodistillation was used to extract EOs from the dried leaves of eight *Eucalyptus* species: *Eucalyptus accedens, Eucalyptus punctata, Eucalyptus robusta*, *Eucalyptus bosistoana, Eucalyptus cladocalyx, Eucalyptus lesouefii, Eucalyptus melliodora* and *Eucalyptus wandoo.* They are assessed by GC/MS and GC/FID and evaluated for antibacterial activity using agar diffusion and broth microdilution techniques against three bacterial isolates (*Haemophilus influenzae*, *Haemophilus parainfluenzae*, *Klebsiella pneumoniae*) and three reference bacteria strains (*Pseudomonas aeruginosa,* ATTC 9027; *Staphylococcus aureus,* ATCC 6538; and *Escherichia coli,* ATCC 8739). Furthermore, the selected twenty-one major compounds and all values of the inhibition zone diameters were subjected to further statistical analysis using PCA and HCA.

**Results:**

The EO yields of the studied *Eucalyptus* species range from 1.4 ± 0.4% to 5.2 ± 0.3%. Among all the species studied, *E. lesouefii* had the greatest mean percentage of EOs. The identification of 128 components by GC (RI) and GC/MS allowed for 93.6% – 97.7% of the total oil to be identified. *1,8*-cineole was the most abundant component found, followed by *α*-pinene, *p*-cymene, and globulol. The chemical components of the eight EOs, extracted from the leaves of *Eucalyptus* species, were clustered into seven groups using PCA and HCA analyses, with each group forming a chemotype. The PCA and HCA analyses of antibacterial activity, on the other hand, identified five groups.

**Conclusion:**

The oils of *E. melliodora*, *E. bosistoana,* and *E. robusta* show promise as antibiotic alternatives in the treatment of otitis media.

**Supplementary Information:**

The online version contains supplementary material available at 10.1186/s12906-021-03379-y.

## Background

The genus *Eucalyptus* L'Herit., native to Australia, belongs to the *Myrtaceae* family and has around 900 species and subspecies [1]. The leaves of over 300 species in this genus produce volatile oil. The oil yields extracted from *Eucalyptus* leaves were reported to range from 0.06% to 7.0% [2, 3]. The pharmaceutical and cosmetic industries have economically exploited less than 20 species of essential oil (EO) rich in 1,8-cineole (> 70%) [4]. Natural medicine has sparked a surge of interest in recent years, particularly those employed to combat microbial agents, as numerous strains have exhibited resistance to pharmacological chemicals [5, 6]. Drug resistance is found in Gram negative bacteria such as *Escherichia coli, Klebsiella pneumoniae and Pseudomonas aeruginosa,* as well as Gram positive bacteria like *Staphylococcus aureus* [7–10]. Drug resistance has led researchers to design novel antimicrobial compounds to treat a variety of human infections [9, 11–14]. Inhalation of EOs extracted from *Eucalyptus* sp. has traditionally been utilized in Tunisian folk medicine to treat respiratory tract illnesses such as pharyngitis, bronchitis, and sinusitis [15]. The ear is connected to the upper respiratory tract by a mucous membrane that connects the nose and throat. *Streptococcus pneumoniae, Haemophilus influenza, Moraxella catarrhalis, Staphylococcus aureus, Haemophilus parainfluenzae, Escherichia coli, Pseudomonas aeruginosa,* and *Klebsiella pneumoniae* have all been found to invade the mucous membrane [16–21]. A variety of respiratory diseases have been associated with these bacterial strains, including acute otitis media (AOM), sinusitis, asthma, and pneumonia [17–21]. Furthermore, several of these bacterial strains, including *P. aeruginosa* and *S. aureus*, as well as *K. pneumoniae* and other microorganisms, are responsible for otitis externa [22]. Every year, the AOM affects over 11% of the world's population (about 700 million individuals) [23]. The majority of them (51%) are children under the age of five [24]. It's worth emphasizing that 31 million AOM patients, including more than 7 million children per year, are at risk of developing chronic suppurative otitis media (CSOM) [25]. Hearing loss can occur in more than half of CSOM patients [26, 27]. Although EOs derived from numerous *Eucalyptus* species have been shown to have antibacterial, antiviral, antioxidant, anti-inflammatory, and antiasthmatic activities [28–30], Few studies have explored the antibacterial activities of EOs against otitis pathogens. We described and investigated the biological activity of EOs isolated from the leaves of 60 *Eucalyptus* species collected from six arboreta in Tunisia in earlier works [7, 31–39]. The aim of the present study is to determine the variability of the yield, the chemical composition, and the antibacterial activities of EOs extracted from leaves of 8 *Eucalyptus* species. The antibacterial properties of microbial strains responsible for otitis are of special interest.

## Methods

### Plant material

We used clean mature leaves from eight species of *Eucalyptus* L'Hér. collected in June, 2017 from the following two regions: i) *Eucalyptus accedens* Fitzg., *Eucalyptus robusta* Sm. and *Eucalyptus punctata* DC. acclimated in Choucha arboretum and located in Sejnane region (37°03′23″N, 9°14′18″E) in the North West of Tunisia, which belongs to the humid inferior bioclimatic stage with mild winter; ii) *Eucalyptus melliodora* A.Cunn. ex Schauer, *Eucalyptus lesouefii* Maiden, *Eucalyptus cladocalyx* F. Muell, *Eucalyptus bosistoana* F. Muell., and *Eucalyptus wandoo* Blakeley were collected from the Mjez Elbab arboretum in the North West of Tunisia (36°38′55″N, 9°36′45″E), which belong to the upper semi-arid bioclimatic stage with moderate winter.

The leaves were collected from three *Eucalyptus* trees, dried on an airy basis, protected from light, packed in paper bags, and stored in the shade. Botanical voucher specimens have been deposited at the Herbarium of the Faculty of Pharmacy's Pharmacognosy laboratory (Monastir, Tunisia) under the following numbers: 0173, 0174, 0175, 0176, 0177, 0178, 0179, 180.

### Extraction of essential oils

The EOs were extracted using a standard apparatus specified by the European pharmacopoeia [40] by hydrodistilling 100 g of roughly crushed leaves for 4 h. For each sample, hydrodistillation was carried out in triplicate. The EOs were collected and dried with Na_2_SO_4_ before being stored at + 4 °C until analysis. The EO yield was calculated as a percentage (%) of the dry weight (v/w).

### GC analysis

The EO extracts were analysed subsequently by GC and GC/MS in triplicates. GC analysis was carried out with a Hewlett-Packard 6890 apparatus equipped with FID and apolar HP5 cap. column. The remaining experiment parameters are as follow: the oven temperature (temp.) was programmed at 60 °C for 1 min, rising gradually from 60 °C to 250 °C at 3 °C/min, and then held isothermal at 250° for 3 min; injector temp. at 250 °C; detector temp. at 280 °C, carrier gas, N_2_ (1.2 mL/min). For each sample, 1μL (10% EO, in purified hexane) was injected for analysis. The relative concentration was calculated using software HP chemstation, which allows assimilating the percentages of the peak areas to the percentages of the various constituents. Retention indices (RI) were determined relatively to the retention time (t_R_) of a series of n alkanes (C_9_-C_28_).

### GC/MS analysis

The EOs were analysed with a Hewlett-Packard 5890 series II apparatus equipped with a 5972 mass selective detector and an apolar HP5 column (30 m × 0.32 mm i.d., film thickness of 0.25 μm). Helium was used as a carrier gas. The mass spectrometer operating conditions were: ionisation voltage, 70 eV; ion source, 230°. The GC analysis was carried out as described above (see GC Analysis).

### Compound identification

The identification of the compounds was based on the comparison of their RI (determined relatively to the t_R_ of n-alkanes (C_9_-C_28_)) and their mass spectra with those of authentic compounds by means of *NBS75K.L.* and *Wiley 275* databases, as well as with literature data [41].

### Antibacterial testing

#### Bacterial strains

In this study, three clinical bacterial isolates (*H. influenzae*, *H. parainfluenzae*, and *K. pneumonia*) were used, as well as three ATCC bacteria: *P. aeruginosa* (ATTC 9027), *S. aureus* (ATCC 6538), and *E. coli* (ATCC 8739). The Microbiology and Immunology Laboratory (EPS Farhat Hachad, Sousse, Tunisia) generously contributed the clinical strains, whereas the ATCC strains were obtained from the culture collection of the Laboratory of Transmissible Diseases and Biologically Active Substances, Faculty of Pharmacy, Monastir, Tunisia.

#### Kirby Bauer paper method

Using bacterial inoculums of 0.5 McFarland and Mueller Hinton (MH) enriched with 5% sheep blood, the antibacterial activity of several EOs was assessed using a paper-disc agar diffusion method. The MH medium for *P. aeruginosa*, *E. coli*, and *S. aureus*, on the other hand, was not enriched. Briefly, 10 μL of each EO was impregnated into absorbent discs (Whatman disc N°3, 6 mm diameter) and then deposited on the surface of infected plates (90 mm). Gentamicine® (10 g/disc) positive control discs were included in each plate. The inhibition zone diameter (izd) was measured and represented in mm after 24 h of incubation at 37 °C.

The results were interpreted as follows: i) not sensitive or no inhibitory effect (-) for izd less than 8 mm; ii) sensitive ( +) or mild inhibitory effect for izd between 8 and 14 mm; iii) very sensitive or moderate inhibitory effect (+ +) for izd between 14 and 20 mm; iv) extremely sensitive or strong inhibitory effect (+ + +) for izd greater than 20 mm [42, 43]. All of the tests were carried out in triplicate, and the results were expressed as mean ± standard errors of mean.

#### Determination of MIC and MBC

The minimum inhibitory concentration (MIC) was determined using the micro-well dilution method according to the National Committee for Clinical Laboratory Standards [44]. An overnight incubated culture (37 °C) of each tested bacterial strain was prepared by adjusting the turbidity of each bacterial culture to reach an optical density of 0.5 McFarland standards. One hundred microliters from each EO diluted in DMSO (10%), initially prepared at a concentration of 931 mg/mL, were added into the third well, followed by two-fold serial dilutions in MH broth medium until the 12^th^ well. Subsequently, 80 μL of MH, 10 μL of the inoculum, and 10 μL of 0.02% resazurin solution were added into each well. The skipped first and the second wells were reserved for negative and positive controls, respectively. Negative control well contained bacteria in the MH broth medium whereas, positive control well contained bacteria in MH broth medium and 10 μg/ mL of Gentamicin® antibiotics.

After incubation for 24 h at 37 °C, the bacterial growth was characterized by color change from blue to pink. The MIC was defined as the lowest concentration that completely inhibits visible cell growth after incubation at 37 °C (blue colored well) for 24 h. To determine the minimum bactericidal concentration (MBC), 10 μL of each culture medium with no visible growth were removed and inoculated in MH plates. After incubation for 18-24 h at 37 °C, the number of surviving organisms was determined. MBC was defined as the lowest concentration at which 99.9% of the bacteria culture were killed [7]. As for all analyses, the experiments were performed in triplicate.

### Statistical analysis

We carried out the analysis of variance (ANOVA test) to compare: i) the EO yields among different *Eucalyptus* species; ii) the quantitative content of chemical components among different *Eucalyptus* species; iii) izd values obtained during the antibacterial analysis among different EOs and among the used bacterial strains. The significance of the difference between means was determined at *p* < 0.05 using Duncan's multiple range test. To evaluate whether the identified EO constituents are a reflection of the chemical and biological activities, the detected 21 chemical compounds in the EO samples (with contents ≥ 2.1% in at least one species) and all theie izd values were subjected to PCA and HCA analyses using IBM SPSS Statistics for Windows, Version 23.0 (Armonk, NY: IBM Corp).

## Results

### Oil Yields

The average EO yields for eight Eucalyptus species ranged from 1.4% ± 0.4 for *E. robusta* Sm. To 5.1 ± 0.4% and 5.2 ± 0.3% for *E. cladocalyx* F. Muell. and *E. lesouefii* Maiden., respectively (Table 1).

**Table 1:** Classification by means of Duncan’s Multiple Range Test of the Average Essential Oil Yields of Eight *Eucalyptus* Species harvested in June in 2017

Eucalyptus species	Yield [%]
*E.accedens*	2.0 ± 0.8(a)^a^
*E.bosistoana*	3.9 ± 0.3(c)
*E.cladoalyx*	5.1 ± 0.4(d)
*E.lesouefii*	5.2 ± 0.3(d)
*E.melliodora*	3.3 ± 0.7(b)
*E.punctata*	1.4 ± 0.4(a)
*E.robusta*	1.7 ± 0.1(a)
*E.wandoo*	2.0 ± 0.1(a)

^a^Yields with different letters in parentheses differ significantly by Duncan’s multiple range test (*p* < 0.05)

The EO yields from three distinct trees revealed that they differed considerably (*p* < 0.05) between species. Four non-overlapping groups of EOs were discovered using the Duncan multiple range test.

### Chemical composition of the tested EOs

The EOs were chromatographically analyzed using GC (RI) and GC (MS), resulting in the identification of 128 compounds (Table 2 Suppl.), accounting for 93.6% – 97.3% of the total oil content. These compounds were further divided into 15 classes (Table 2 Suppl.).

The major class was constituted by the monterpenic oxides (27.4% – 66.3%), with 1,8-cineole having the highest proportion (28.1% – 66.3%) (Table 2). The second major class was constituted by the monterpens hydrocarbons (4.6% – 51.2%), with *α*-pinene and *p*-cymene as prominent constituents (3.9% – 38.2% and 0.4% – 35.8%, respectively).

**Table 2:** Chemical Composition of the Essential Oils Extracted from Leafs of Eight *Eucalyptus* Species with content ≥ 1.0%

Compound class and Name	RI^a^	Content [%]							
		*E. accedens*	*E. bosistoana*	*E. cladocalyx*	*E. lesouefii*	*E. melliodoa*	*E. robusta*	*E. punctata*	*E. wandoo*
***Monoterpenes hydrocarbons***									
α-Pinene	932	38.2	10.8	3.9	12.8	9.2	15.1	4.2	6.5
Camphene	952	0.1	0.1	tr^b^	0.1	0.2	1.8	0.3	0.2
*β*-Pinene	976	-^c^	0.3	tr	10.9	0.1	0.1	5.4	0.1
*α*-Phellandrene	1 005	2.2	0.2	tr	0.1	0.2	0.2	0.2	tr
*p*-Cymene	1 024	8.6	4.0	0.4	7.7	0.4	11.8	28.7	35.8
*γ*-Terpinene	1 057	0.1	0.1	0.1	0.3	tr	0.3	0.1	3.9
***Monoterpene oxides***									
1.8-Cineole	1 030	28.1	52.7	39.2	38	66.3	26.5	20.7	37.7
***Monterpene ketones***									
Cryptone	1 186	0.1	0.6	0.3	0.8	-	-	8.4	0.2
Verbenone	1 227	0.2	0.5	0.1	0.3	-	0.6	1.5	0.2
***Monoterpene aldehydes***									
Citronellal	1 157	0.1	0.1	0.4	1	0.1	3.5	0.2	tr
Cuminaldehyde	1 239	tr	tr	tr	0.1	tr	0.2	2.1	tr
Phellandral	1 274	-	tr	-	tr	tr	-	1.1	tr
***Monterpene alcohols***									
*D*-fenchyl alcohol	1 113	0.1	0.1	0.1	0.2	0.4	2.2	0.3	0.2
*trans*-Pinocarveol	1 138	2.3	3.1	2	3.2	4	5.3	4.2	2.2
*endo*-Borneol	1 162	0.5	0.9	0.3	0.4	0.7	6.0	1.1	0.4
Borneol	1 171	tr	tr	0.1	1.8	tr	0.4	0.3	tr
Terpinen-4-ol	1 176	0.4	0.2	0.3	3.3	0.3	0.3	0.4	1.1
*α*-Terpineol	1 191	0.5	0.9	0.2	1.2	1.6	6.7	0.5	1.2
*p*-Cymen-8-ol	1 196	0.2	0.1	tr	-	0.1	0.4	3.0	0.1
Cuminol	1 290	-	tr	tr	0.1	tr	0.2	1.1	0.1
**Sesquitepene hydrocarbons**									
*α*-Cubebene	1 346	0.1	-	0.1	-	1.7	-	-	-
Aromadendrene	1 438	0.2	7.3	8.7	0.4	0.6	0.2	0.1	0.1
Alloaromadendrene	1 460	0.1	1.5	1.3	0.4	0.3	0.1	0.1	tr
Ledene	1 492	tr	tr	1.6	tr	-	tr	0.1	tr
***Sesquiterpene alcohols***									
epiglobulol	1 552	tr	0.9	2.3	0.1	0.1	0.3	0.1	tr
Spathulenol	1 577	2.5	4.1	0.2	4.6	0.5	0.2	1.6	-
Globulol	1 584	2.4	2.3	12.7	0.8	1.2	1.4	-	0.1
Viridiflorol	1 591	1	0.7	2.6	0.1	0.2	0.3	0.1	0.2
Rosifoliol	1 612	0.2	0.4	1.7	tr	tr	5.2	0.1	tr
Hinesol	1 642	0.6	0.1	0.1	-	0.1	0.1	-	1.2
*β*-Eudesmol	1 645	0.7	0.2	0.5	1.1	-	0.1	tr	0.3
***Sesquiterpene oxide***									
Caryophyllene oxide	1 583	-	-	-	-	-	-	1.5	-
*Aliphatic esters*									
Methyl amyl acetate	900	tr	tr	8.9	tr	tr	tr	tr	tr

^a^RI: Retention index determined on HP5 cap. Column. ^b^ tr: Trace (< 0.1); ^c^ Not detected

The sesquiterpenic alcohols were the third most common class (2.4% – 21.6%), with globulol (0.0 – 12.7%), rosifoliol (trasse – 5.2%), and spathulenol (0.0 – 4.6%) being the most common. Monoterpenic alcohols (3.4% – 23.0%) are the fourth major class, with *α*-terpineol (0.2% – 6.7%), *endo*-borneol (0.3% –6.0%), and trans-pinocarveol (2.0% – 5.3%) are the most prominent components.

Squiterpene hydrocarbons (0.4% – 14.4%), with aromadendrene as a significant ingredient (0.1% – 8.7%), were the class with the sixth largest content. Monoterpenic ketones (0.6% – 12.2%) were the sixth main class, with cryptone (0.0 – 8.4%) being a prominent element (0.0 – 8.4%).

The aliphatic esters (tr – 8.9%), which include methyl amyl acetate, are the seventh significant class. The monterpene aldehydes (0.1% – 3.7%) were the eighth main class, with citronellal (tr – 3.5%) being a prominent element. Minor compounds having a mean proportion of less than 1.1% made up the rest of the classes.

The monoterpenic oxide 1,8-cineole (66.3%) represented the highest percentage in EO isolated from *E. melliodora* leaves, as well as a comparatively significant amount of the monterpenic aldehyde trans-pinocarveol and the monoterpenic hydrocarbons *α*-pinene (4% and 9.2%, respectively). Many additional elements, such as *p*-cymene, *β*-pinene, cryptone, and cuminal, were comparatively low.

*E. accedens* EO had the highest mean percentage of monoterpenic hydrocarbons *α*-pinene (38.2%), whereas *E. wandoo* EO had the highest mean percentage of monoterpenic hydrocarbons *p*-cymene and *γ*-terpinene (37.7% and 3.9%, respectively).

The monoterpenic hydrocarbon *β*-pinene (10.9%), the monoterpenic alcohol trepinen-4-ol (3.3%), and the sesquiterpenic alcohol spathulenol (4.6%) were found in large amounts in *E. lesouefii* EO, while *p*-cymene, *α*-pinene, and *1,8*-cineole were found in modest amounts (7.7%, 10.8%, and 38%, respectively).

*E. pimpiniana* EO had the largest concentrations of the monterpinc ketone cryptone (8.4%), monoterpenic alcohol *p*-cymen-8-ol (3.0%), and monoterpenic aldehyde cuminaldehyde (2.1%), as well as a high mean proportion of the monoterpenic hydrocarbons p-cymene and the monoterpene alcohol *trans*-pinocarveol (28.7% and 4.2%, respectively).

The monoterpenic alcohols *endo*-borneol (6.0%), *α*-terpineol (6.7%), *trans*-pinocarveol (5.3%), sesquiterpenic alcohol rosifoliol (5.2%), and monoterpenic aldehyde citronellal (3.5%) were found in the highest concentrations in *E. robusta* EO, and a relatively high amount of the monoterpenic hydrocarbons *α*-pinene (15.1%) and *p*-cymene (11.8%).

In *E. cladocalyx* EO, the highest mean percentages of sesquiterpenic alcohols globulol (12.7%), epiglobulol (1.7%), viridiflorol (2.3%), sesquiterpenic hydrocarbons aromadendrene (8.7%), and ester methyl amyl acetate (8.9%) were detected, but *β*-pinene, *p*-cymene, and *α*-pinene were very poor.

*E. bosistoana* EO was relatively rich in *1,8*-cineole and *α*-pinene with comparative mean percentages as those observed in *E. pimpiniana*.

### Principal Component (PCA) and Hierarchical Cluster (HCA) analyses

**Fig. 1:**
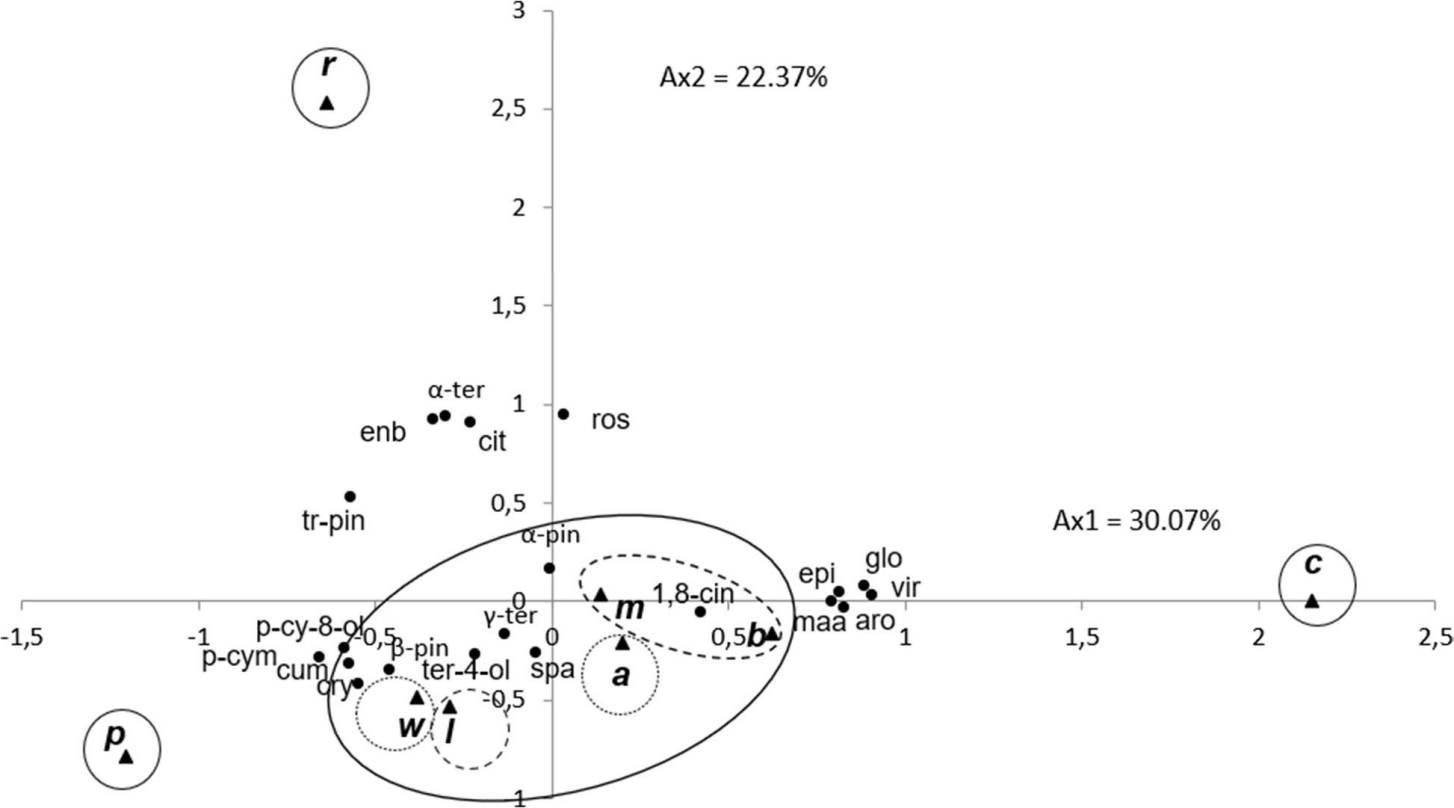
PCA of twenty components for the leaf essential oils of eight Tunisian *Eucalyptus* species. For the abbreviation of the *Eucalyptus* species (▲): **a**: *E. accedens*; **b**: *E. bosistoana*; **c**: *E. cladocalyx*;** l**: *E. lesouefei*i; m: *E. melliodora*; p: *E. punctata*; r: *E. robusta*; W: *E. wandoo*

**Fig. 2:**
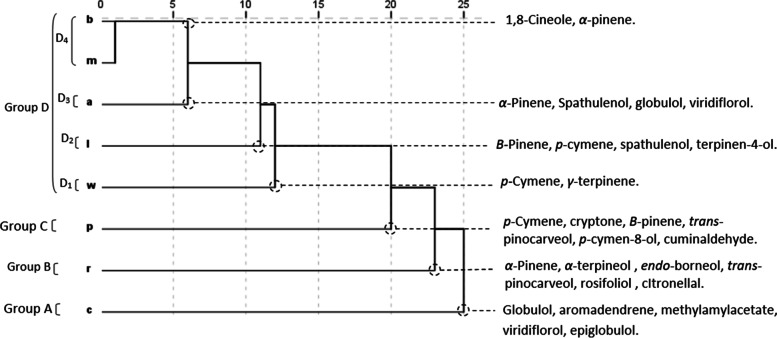
Dendrogram obtained by hierarchical cluster analysis based on the Euclidean distance between groups of leaf essential oils of eight Tunisian *Eucalyptus* species. Components that characterize the major subgroups, considered as chemotypes, are indicated. For the abbreviation of the *Eucalyptus* species (▲), see legend Fig. 1

To evaluate whether the identified EO components may be useful in reflecting the chemotaxonomic relationships of the eight *Eucalyptus* species, 21 chemical compounds with a yield greater or equal to 2.1% in at least one species (Table 3) were selected for the PCA (Fig. 1) and the HCA analyses (Fig. 2). The concentrations of these chemical components differed significantly between species (p < 0.05).The HCA analysis identified four groups (A, B, C and D)*,* identified by their EO chemotypes with a dissimilarity of greater than 15%. Group D was further divided into four subgroups (D_1_-D_4_) with a dissimilarity of greater than 5%. The PCA horizontal axis (axis 1) explained 30.07% of the total variance due to the increasing level of the mean percentage of compounds in group A and C species. The variation along the PCA vertical axis (axis 2) (22.37%) was mainly due to the increase in the mean percentage of compounds in group B and their decreasing level in group C and subgroups D_1_ and D_2_, which stand out in both HCA and PCA analyses, forming separate groups and subgroups. Since components of the EOs within the same group were significantly correlated and tend to vary in the same way, we considered each group as a chemotype. Group A is constituted by *E. cladocalyx*, for which the EO content is distinguished from other groups by the highest percentages of sesquiterpenic alcohols globulol (12.7 ± 2.9%)*,* epiglobulol (2.3 ± 0.5%), viridiflorol (2.6 ± 0.6%), the sesquiterpenic hydrocarbons aromadendrene (8.7 ± 0.7%) and the ester methyl amyl acetate (8.9 ± 1.5%), but by the absence of the monoterpenic alcohol *p*-cymen-8-ol, monoterpenic hydrocarbons *p*-cymene and the aldehyde cuminal. On the other hand, *E. robusta,* constituting *Group* B*,* was positively correlated with axis 2 and stood out, forming a separate group in both the HCA and PCA analyses. It was characterized by the highest content in the monoterpenic alcohols *trans*-pinocarveol (5.3 ± 0.2%), *endo*-borneol (6.0 ± 0.3%), *α*-terpineol (6.7 ± 0.3%), aldehyde citronellal (3.5 ± 0.1%), and the sesquiterpenic alcohol rosifoliol (5.2 ± 0.5%). This separation was enhanced further by its poverty in cryptone, *β*-pinene, and terpinen-4-ol. Group C, constituted by E*. pimpiniana*, was negatively correlated with axis 1. The EO of *E. pimpinianais is* characterized by its highest content of cryptone (8.4 ± 1.6%), *p*-cymen-8-ol (3.0 ± 1.2%), and cumianldehyde (2.1 ± 0.6%). It was also close to *E. wandoo* of the subgroup D_1_, likely due to its relative richness in *p*-cymene (28.7 ± 5.7%) and to *E. lesouefii* of the subgroup D_2_ by its richness in *β*-pinene (5.4 ± 2.4%). Both *E. robusta* and *E. pimpiniana* EOs were negatively correlated with axis 1, mainly due to their relative poverty in 1,8-cineole (26.5 ± 0.3 and 20.7 ± 12.2%, respectively). Sub-group D1, constituted by E. wandoo, is characterized by *p*-cymene (35.8 ± 4.3%) and *γ*-terpinene (3.9 ± 1.9%), whereas *E. lesouefii*, constituting the subgroup D2, is characterized by *β*-pinene (10.9 ± 0.0%), spathulenol (4.6 ± 0.2%) and terpine-4-ol (3.3 ± 0.0%). Subgroup D3, constituted by *E. accedens,* is characterized by α-pinene (38.2 ± 13.7%) and a relatively high content of spathulenol, globulol, and viridiflorol. Subgroup D_4_, formed by *E. melliodora* and *E*. *bosistoana* oils, is characterized by *1,8*-cineole (66.3 ± 1.0% and 52.7 ± 8.6%, respectively). The separation between the two species was mainly due to the richness of *E. bosistoana* in aromadendrene (7.3 ± 3.7%), against 0.6 ± 0.2% in *E. melliodora*. The statistical analysis revealed significant variability in the EOs among the *Eucalyptus* species. The HCA and PCA analyses identified seven groups and subgroups, yet 19 major chemical components were identified; each group constituted a chemotype.

**Table 3:** Content [%] of the 20 Compounds selected for the Principal Component and the Hierarchical Cluster Analyses in the essential Oils Extracted from the Leafs of eight Eucalyptus species

Compounds	Abbreviation	Content[%]							
		*E. accedens*	*E. bosistoana*	*E. cladocalyx*	*E. lesouefii*	*E. melliodoa*	*E. punctata*	*E. robusta*	*E. wandoo*
*α*-Pinene	α-pin	38.2 ± 13.7	10.8 ± 4.5	3.9 ± 0.8	12.8 ± 0.2	9.2 ± 0.8	4.2 ± 2.4	15.1 ± 2.0	6.5 ± 0.4
*Β*-pinene	Β-pin	-	0.3 ± 0.2	tr	10.9 ± tr	0.1 ± tr	5.4 ± 2.4	0.1 ± tr	0.1 ± tr
*p*-cymene	p-cym	8.6 ± 2.9	4.0 ± 0.6	0.4 ± 0.1	7.7 ± tr	0.4 ± 0.5	28.7 ± 5.7	11.8 ± tr	35.8 ± 4.3
*1,8*-cineole	1,8-cin	28.1 ± 6.1	52.7 ± 8.6	39.2 ± 5.0	38.0 ± 0.6	66.3 ± 1.0	20.7 ± 12.2	26.5 ± 0.3	37.7 ± 4.9
*γ*-Terpinene	γ-ter	0.1 ± tr	0.1 ± tr	0.1 ± tr	0.3 ± tr	tr	0.1 ± 0.1	0.3 ± tr	3.9 ± 1.9
trans-Pinocarveol	tr-pin	2.3 ± 0.9	3.1 ± 0.3	2.0 ± tr	3.2 ± tr	4.0 ± tr	4.2 ± 1.6	5.3 ± 0.2	2.2 ± 0.4
CItronellal	cit	0.1 ± tr	0.1 ± tr	0.4 ± tr	1.0 ± tr	0.1 ± tr	0.2 ± tr	3.5 ± 0.1	tr
*endo*-Borneol	enb	0.5 ± 0.2	0.9 ± 0.1	0.3 ± tr	0.4 ± tr	0.7 ± tr	1.1 ± 0.4	6.0 ± 0.3	0.4 ± 0.1
Terpinen-4-ol	Ter-4-ol	0.4 ± 0.1	0.2 ± tr	0.3 ± tr	3.3 ± tr	0.3 ± tr	0.4 ± 0.2	0.3 ± 0.4	1.1 ± 0.4
Cryptone	cry	0.1 ± 0.1	0.6 ± 0.1	0.3 ± tr	0.8 ± tr	-^b^	8.4 ± 1.6	-	0.2 ± tr
*α*-Terpineol	α–ter	0.5 ± tr	0.9 ± 0.1	0.2 ± 0.2	1.2 ± tr	1.6 ± tr	0.5 ± 0.2	6.7 ± 0.3	1.2 ± 0.3
*p*-Cymen-8-ol	p-cy-8-ol	0.2 ± tr	0.1 ± tr	tr	-	0.1 ± tr	3.0 ± 1.2	0.4 ± tr	0.1 ± tr
Cuminaldehyde	cum	tr	tr	tr	0.1 ± tr	tr	2.1 ± 0.6	0.2 ± 0.2	tr
Aromadendrene	aro	0.2 ± 0.2	7.3 ± 3.7	8.7 ± 0.7	0.4 ± tr	0.6 ± 0.2	0.1 ± 0.1	0.2 ± 0.3	0.1 ± tr
Epiglobulol	epi	tr	0.9 ± 2.3	2.3 ± 0.5	0.1 ± tr	0.1 ± tr	0.1 ± tr	0.3 ± tr	tr
Spathulenol	spa	2.5 ± 3.2	4.1 ± 5.1	0.2 ± tr	4.6 ± 0.2	0.5 ± 0.1	1.6 ± 0.4	0.2 ± 0.3	-
Globulol	glo	2.4 ± 0.3	2.3 ± 0.8	12.7 ± 2.9	0.8 ± tr	1.2 ± 0.4	-	1.4 ± 0.1	0.1 ± tr
Viridiflorol	vir	1.0 ± 0.2	0.7 ± 0.1	2.6 ± 0.6	0.1 ± tr	0.2 ± tr	0.1 ± tr	0.3 ± tr	0.2 ± tr
Rosifoliol	ros	0.2 ± 0.1	0.4 ± 0.4	1.7 ± 0.4	tr	tr	0.1 ± 0.1	5.2 ± 0.5	tr
Methyl amyl acetate	maa	tr	tr^a^	8.9 ± 1.5	tr	tr	tr	tr	tr

^a^tr: Trace (< 0.1%).^b−^: Not detected

### Antibacterial testing

**Fig. 3:**
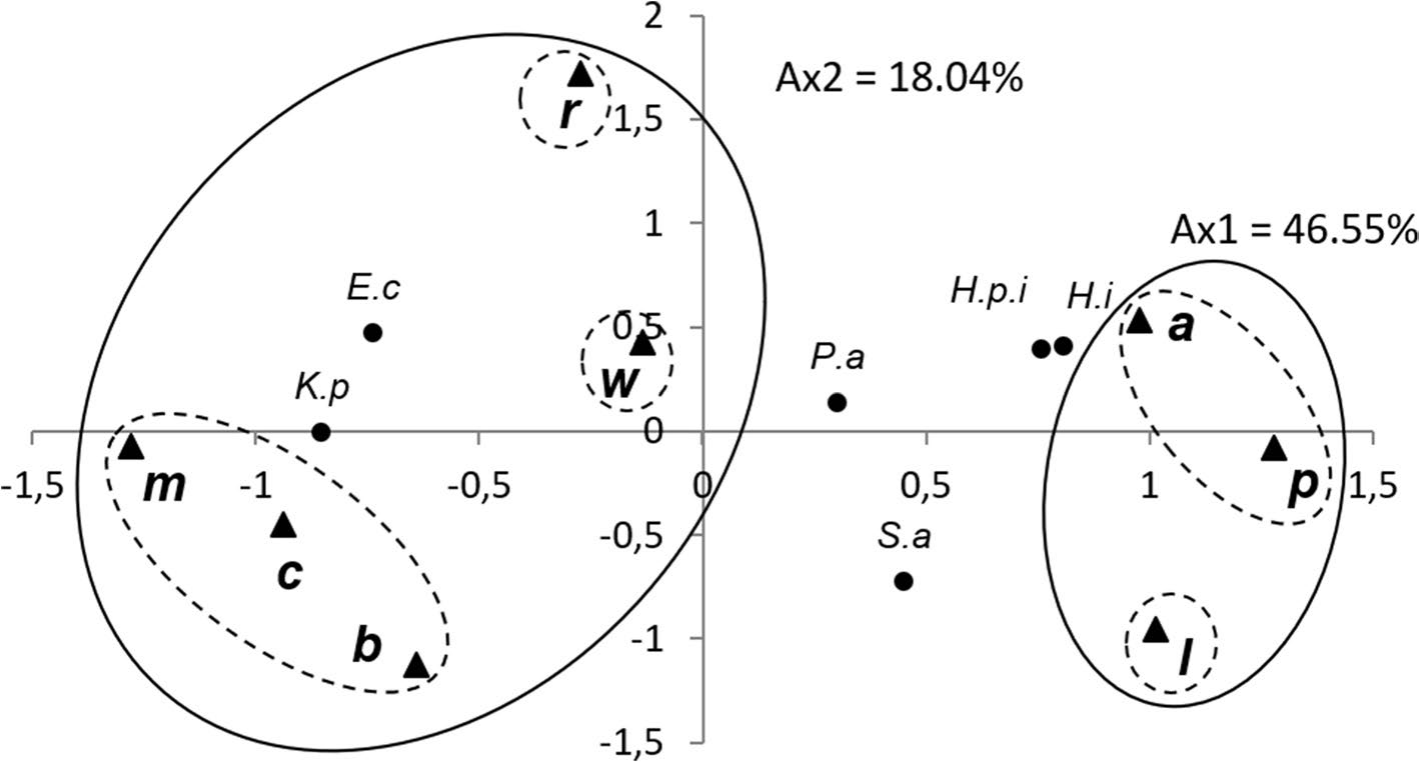
PCA of the antibacterial activities of leaf essential oils of eight Tunisian *Eucalyptus* species. For the abbreviation of the *Eucalyptus* species (▲), see legend Fig. 1.*) *E.c: Echerichia coli; k.p: Klebsiella pneumoniae; P.a: Pseudomonas aeruginosa; S.a: Staphylococcus aureus; H.i: Haemophilus influenzae; H.p.i: Haemophulis parainfluenzae*

**Fig. 4:**
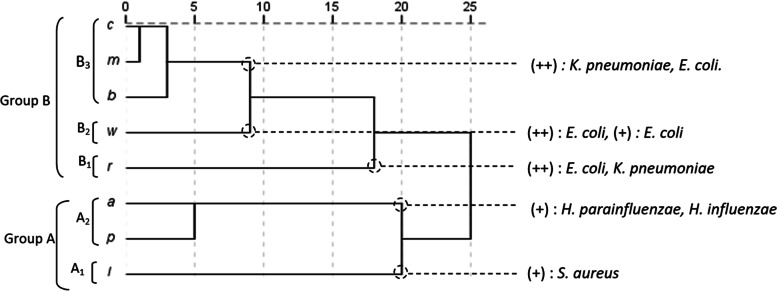
Dendrogram obtained by hierarchical cluster analysis based on the Euclidean distance between groups of the antibacterial activities of EOs of eight Tunisian *Eucalyptus* species. *) For the abbreviation of the *Eucalyptus* species, see legend Fig. 1

The EOs were tested for their putative antibacterial activity against six bacterial strains (Table 4). The results showed that, with the exception of Gram negative *P. aeruginosa*, the majority of these bacterial strains were sensitive to the tested EOs. The Gram negative *E. coli* was sensitive to EOs extracted from *E. robusta*, *E. melliodora*, and *E. wandoo*, but it was resistant to EO extracted from *E. punctata*. Moreover, the EO extracted from *E. melliodora* possessed the best activity against the Gram negative *K. pneumoniae*, followed by those extracted from *E. bosistoana* and *E. robusta*. In order to evaluate the relationship between the EOs extracted from the eight Eucalyptus species and their antibacterial activities, all the mean values of izd were subjected to PCA and HCA analyses. Antibacterial activities of the tested EOs showed a significant difference between *Eucalyptus* species and bacterial strains (p < 0.05). The PCA horizontal axis (axis 1) explained 46.55% of the total variance, while the vertical axis (axis 2) explained a further 18.4% (Fig. 3). The HCA analysis identified two EO groups (A' and B') distinguished by antibacterial activity and a dissimilarity greater than or equal to 20 (Fig. 4). With a dissimilarity of > 5, group A was further subdivided into two subgroups (A'1 and A'2), whereas group B was further subdivided into three subgroups (B'1, B'2, and B'3). Axis 1 divides group A from group B, while axis 2 divides group A into two subgroups and group B into three subgroups. Group A’, constituted by *E. accedens*, *E. punctata* and *E. lesouefii*, forms a deep dichotomy in the HCA analysis and a clearly separated group in the PCA analysis. These species were characterized by their lowest activity against *K. pneumoniae* and *E. coli* (6.0 ± 0.0 mm ≤ izd ≤ 12.3 ± 3.8 mm). *E. lesouefii* of the subgroup A’1 showed the highest activity against the Gram positive *S. aureus* (13.3 ± 1.2 mm, izd). *E. accedens* and *E. punctata*, belonging to the subgroup A’2, were more active against *H. parainfluenzae* and *H. influenzae*, respectively. *E. robusta*, belonging to subgroup B’1, showed similar activity to the reference Gentamicine® against *E. coli* and had moderate activity against *K. pneumoniae*. Subgroup B’2, constituted by *E. wandoo oil*, was characterized by a mild inhibitory effect against all the tested bacterial strains, except *E. coli*. *Eucalyptus* species, belonging to the subgroup B'3, showed relatively moderate activity against *K. pneumoniae*. However, *E. melliodora* and *E. cladocalyx* showed promising activity against *E. coli*. Altogether, the tested EOs were less active than the Gentamicine®. The MIC results showed that the EO, rich in globulol, epiglobulol, methyl amyl acetate and aromadendrene, extracted from *E. cladocalyx*, showed the lowest MIC value against *H. influenza* (Table 5). The second lowest MIC was shown for *E. robusta* and *E. melliodora* against *E. coli* (14.06 μg/mL, 25.97 μg/mL, respectively). These results were further confirmed by the disc diffusion method. The highest MIC against *S. aureus* and *E. coli* was shown for EOs extracted from *E. lesouefii* and *E. accedens*. The highest MIC against *P. aeruginosa* and *H. influenzae* was shown for EOs extracted from *E. bosistoana* and *E. lesouefii* (415.50 mg/mL), whereas the lowest MIC against the same bacterial strain was shown for EO extracted from *E. wandoo* (51.94 mg/mL). These findings were in contradiction to the results observed using the disc diffusion method. According to the classification of Schaechter et al. (1999) and Dramane et al. (2010) [45, 46], all the tested oils were considered Bactericidal against the tested bacterial strains (MBC/MIC ≤ 4). However, the best bactericidal activity against *P. aeruginosa*, *K. pneumoniae* and *H. parainfluenzae* was observed for EOs extracted from *E. punctata* and *E. bosistoana*. Moreover, EOs extracted from *E. lesouefii*, *E. accedens* and *E. melliodora* showed promising antibacterial activity against *S. aureus*, whereas EO extracted from *E. cladocalyx* oil showed high antibacterial activity against both *H. influenzae* and *H. parainfluenzae*.

**Table 4:** Diameter of the inhibition of the inhibition of ear infection bacterial growth by individual essential oils and by the antibiotic (Gentamicin)

*Eucalyptus* Species oils	Bacterial Strains					
	Gram-negative					Gram-positive
	*E.coli*	*H. influenzae*	*H.parainfluenza e*	*K. pneumoniae*	*P.aeruginosa*	*S.aureus*
*E. accedens*	12.3 ± 3.8^b*)^	1tr ± 1.0^ab^	12.7 ± 2.5^b^	7.0 ± 1.7^a^	6.3 ± 0.6^a^	9.3 ± 0.6^a^
*E. bosistoana*	13.7 ± 1.5^bc**)^	6.0 ± tr^a^	7.3 ± 1.5^ab^	16.0 ± 1.7^bcd^	6.0 ± tr^a^	10.7 ± 4.0^ab^
*E. cladocalyx*	15.7 ± 3.2^bcd^	6.0 ± tr^a^	6.3 ± 0.6^a^	15.0 ± 4.4^bc^	6.0 ± tr^a^	8.0 ± 2.6^a^
*E.lesouefii*	1tr ± 2.0^ab^	9.3 ± 1.2^ab^	9.3 ± 2.1^ab^	8.7 ± 1.2^a^	8.3 ± 0.6^a^	13.3 ± 1.2^b^
*E. melliodora*	19.7 ± 6.7^de^	6.3 ± 0.6^a^	7.0 ± 1.7^ab^	19.7 ± 2.9^ cd^	6.0 ± tr^a^	8.3 ± 2.1^a^
*E. punctata*	6.0 ± tr^a^	11.7 ± 2.1^b^	10.7 ± 3.5^ab^	7.0 ± tr^a^	6.0 ± tr^a^	9.7 ± 3.2^a^
*E. robusta*	20.7 ± 1.5^de^	1tr ± 1.0 ^ab^	1tr ± 2.6^ab^	16.0 ± 4.6^bcd^	7.7 ± 1.5^a^	6.3 ± 0.6^a^
*E. wandoo*	18.3 ± 0.6^cde^	9.0 ± 1.0 ^ab^	9.3 ± 0.6^ab^	12.0 ± 1.7^ab^	6.0 ± tr^a^	9.0 ± 1.0^a^
Gentamicin	24.2 ± 2.3^e^	31.4 ± 2.1^c^	38.6 ± 2.8^c^	21.5 ± 2.4^d^	26.4 ± 1.6^b^	29.9 ± 1.0^c^

^*)^ Values are means (mm ± MSD) of triplicate determination; ^**)^ Values with different letters differ significantly by Duncan’s multiple range test (*P* < 0.05)

**Table 5:** Minimal inhibitory concentration (MICs), minimal bactericidal concentrations (MBC s), and MBC/MIC ratio for *Eucalyptus* oils

Oils	Bacterial strains																	
	Gram-negative															Gram-positive		
	*Echerichia coli*			*Haemophilus influenzae*			*Haemophilus parainfluenzae*			*Klebsiella pneumoniae*			*Pseudomonas aeruginosa*			*Staphylococcus aureus*		
	MIC	MBC	MBC/MIC	MIC	MBC	MBC/MIC	MIC	MBC	MBC/MIC	MIC	MBC	MBC/MIC	MIC	MBC	MBC/MIC	MIC	MBC	MBC/MIC
*E. accedens*	415.5	-	-	207.7	207.7	1.0	103.9	103.9	1.0	51.9	103.9	2.0	103.9	207.7	2.0	415.5	415.5	1.0
*E. bosistoana*	51.9	103.9	2.0	103.9	207.7	2.0	207.7	207.7	1.0	103.9	103.9	1.0	415.5	415.5	1.0	103.9	207.7	2.0
*E. cladocalyx*	103.9	207.7	2.0	3.2	3.2	1.0	207.7	207.7	1.0	51.9	103.9	2.0	103.9	207.7	2.0	103.9	415.5	4.0
*E. lesouefii*	415.5	-	-	415.5	-	tr	415.5	-	tr	207.7	415.5	2.0	415.5	-	NT	415.5	415.5	1.0
*E. melliodora*	26.0	51.9	2.0	103.9	207.7	2.0	207.7	415.5	2.0	26.0	51.9	2.0	207.7	415.5	2.0	103.9	103.9	1.0
*E. punctata*	103.9	207.7	2.0	103.9	207.7	2.0	207.7	207.7	1.0	103.9	103.9	1.0	103.9	103.9	1.0	103.9	207.7	2.0
*E. robusta*	14.1	26.0	1.8	103.9	207.7	2.0	103.9	103.9	1.0	51.9	103.9	2.0	103.9	207.7	2.0	207.7	415.5	2.0
*E. Wandoo*	103.9	207.7	2.0	51.9	103.9	2.0	51.9	103.9	2.0	51.9	103.9	2.0	51.9	103.9	2.0	51.9	103.9	2.0
Gentamicine	0.6	2.5	4.2	6.0	24.0	4.0	6.0	24.0	4.0	4.0	16.0	4.0	10.0	10.0	1.0	5.0	20.0	4.0

## Discussion

### Oil Yields

The present results showed that *E. cladocalyx* F. Muell*.* grown in Mjez elbab arboretum (North East of Tunisia), was much richer in EO than those obtained from Algeria (0.49%), Morocco (0.30 – 0.80%) [47–49], and even from another Tunisian location (Zerniza arboretum, region of Sejnene, North West of Tunisia and Sidi Smail arboretum, Region of Monastir) (1.9 ± 0.1 – 3.06%) [33, 50]. Additionally, *E. melliodora* leaves were also richer in EOs than those collected from Morocco (1.68%) [51] and Australia (0.08%) [52]. However, the Iranian provenance demonstrated similar results with the mean EO yield varying from 2.6 to 3.9% [53]. Compared with the results obtained by other studies, Tunisian plantation of *E. robusta* was much richer in EOs than those from Congo (0.13%) [54], Australia (as tr) [55], Brazil (0.2 – 0.34%) [56, 57], China (0.17%) [58], and Algeria (0.6%) [59]. Moreover, the EO yield of *E. punctata* leaves (1.4 ± 0.4%) showed similar yields as those reported in Australia, Morroco, and Algeria (1.3 ± 0.6 – 1.57%) [47, 51, 60], whereas those from Uruguay provenance showed less EO yield (0.33%) [61]. Our findings also revealed that the leaves of *E. accedens* have a higher EO yield than those from Australian plantations (0.9%) [62]. In addition, *E. bosistoana* from Tunisia was much richer in EOs (3.9 ± 0.3%) than those obtained from Morocco, Algeria and Portugal (0.6 – 1.8%) [51, 63, 64]. These variations could be linked to the environmental influence on *Eucalyptus* EO biosynthesis [65–67]. It is worth noting that the EO yields of *E. Wandoo* and *E. lesouefii* have not been studied previously.

### Chemical composition of the tested essential oils

The chemotaxonomic variation shown in the results could be attributed to exogenous factors such as precipitation, temperature, light, soil type, altitude light etc., and to endogenous ones, related mainly to the anatomical, physiological and genetic characteristics of the plant, controlling the EO biosynthesis. Furthermore, the environment may influence the DNA of the aromatic plants, resulting in different genotype [68]. It was reported that the chemical composition of both *E. camaldulensis* and *E. loxophleba* EOs was dependent on their physiological stage, which was dependent on genetic factors and on external factors such as soil moisture conditions [67, 69]. Moreover, the correlation between the EOs' chemical composition and the genetic diversity of many aromatic plant species has been demonstrated by a number of researchers who point out the chemotype / genotype association [70, 71].

*E. bosistoana* EO has similar major compounds to those obtained by Zrira et al.(1992) [51] with a small difference in their mean percentages. However, the studies of Faria et al. (2011) [64] and Bouzabata et al. (2014) [63] noted the presence of other major components such as *α*-terpineol (6.9%), limonene (4.5%), *p*-cymene (32.0% – 39.5%), cryptone (11.5% – 15.6%), and *α*-pinene (11.6% – 12.1%). *E. punctata* and *E. melliodora* from Morocco and Algeria were much richer in 1,8-cineole (44.0% and 58.2%, respectively) and in *α*-pinene (19.6% and 7.7%, respectively) [47, 51]. Our findings on their major EOs components agree with the results obtained by Southwell (1973) [60] and Bignell et al. (1997d) [55], but our results disagree with those obtained by Filomeno et al. (2017) [56], which demonstrated the presence of a relatively high content in *1,8*-cineole (55.6%), *α*-pinene (27.2%), *α*-phellandrene (6.8%), and a low mean percentage of *p*-cymene (3.0%) in the Brazialian *E. punctata* EO [72]. *E. wandoo* from Algeria was much poorer in *1,8*-cineole and *p*-cymene (14.9% and 9.0%, respectively), but it was distinguished by its high content of benzaldehyde (32.3%) [47]. The latter compound was not detected in our investigation. *E. robusta* from Algeria and Indonesia [59, 73] were characterized by a higher content of 1,8-cineole (50.0% and 55.8%, respectively) and *α*-pinene (22.2% and 37.05%, respectively) than that obtained from Tunisia, whereas a similar percentage of *1,8*-cineole was observed in the China provenance [58] with a higher content of *α*-pinene (30.18%). Moreover, different main compounds such as myrtenal, pinocarvone, isobicyclogermacral and *α*-phellandrene were detected in those from Australia, Congo and Brazil [54–56].The *E. accedens* EO from Australia was much richer in *1,8*-cineole (71.5%), trans-pinocarveol (15.8%), and aromadendrene (7.3%) than the one obtained in our study, but the Tunisian *E. accedens* EO was richer in *α*-pinene (38.2 ± 13.7%) than the Australian one (9%) [62]. Different main compounds were detected in *E. cladocalyx* from Zerniza arboretum (North of Tunisia), such as *α*-terpineol (18.0 ± 4.5%) and boroneol (24.8 ± 4.1%) [74], but we noted a relatively high mean percentage of methyl amyl acetae (8.9 ± 1.5%) in samples from Mjez elbeb arboretum and its complete absence in the same species from Zerniza arboretum, which was relatively poor in *1,8*-cineole (3.0 ± 0.0%), globulol (0.3 ± 0.6%) and aromadendrene (0.1%).

The variation in the chemical composition of the EOs could be attributed to environmental factors that affect the biosynthesis of the EOs’ compounds in both quantity and quality [75]. To the best of our knowledge, the chemical composition of *E. lesouefii* EO has not been studied previously.

### Antibacterial testing

Altogether, the antibacterial activity of the EOs displayed considerable variation among the different *Eucalyptus* species oils, but is still much lower than that of the standard antibiotic Gentamicine®. This variability could be attributed to the chemical composition of the leaf oils [76].

The EO extracted from *E. robusta*, rich in the monoterpene aldehyde citronellal, the monterpene alcohols endo-borneol, α-terpineol and the sesquiterpene alcohol rosifoliol, showed the highest activity against *E. coli* and a moderate inhibitory effect against *K. pneumoniae*. The EO extracted from *E. punctata*, characterized by the highest amount of the ketone cryptone, the monterpene aldehyde cuminal, the monoterpene alcohol *p*-cymen-8-ol and monoterpene hydrocarbons p-cymene (28.7 ± 5.7%), showed the lowest inhibition effect against *E. coli* and *K. pneumoniae*, but had the highest activity against *H. influenzae*. The main activity against *E. coli* is likely attributed to the higher content of the components characterizing *E. robusta* EO (monoterpene aldehyde citronellal, the monterpene alcohols *endo*-borneol, *α*-terpineol and the sesquiterpene alcohol rosifoliol), whereas *H. influenzae* was more sensible to EOs rich in cryptone, cuminal, *p*-cymen-8-ol and p-cymene. It was reported by Griffin et al. (1999), that compounds of smaller volume with high hydrogen-bonding capacity interact significantly with water and tend to be active against the Gram negative *E. coli* [77]. It was also reported by the same author that the aldehyde citronellal has low water solubility and was inactive against the same strain. Therefore, we could deduce that the monoterpene alcohols, *endo*-borneol, *α-terpineol,* could be the main compounds responsible for the activity against *E. coli. E. melliodora* oil, characterized by the highest mean percentage of 1,8-cineole, produced the highest antibacterial activity against *K. pneumoniae* and a medium inhibitory effect against *E. coli*. In *E. bosistoana* EO, this activity has decreased, as evidenced by a lower mean percentage of *1,8*-cineole and a higher content of spathulenol. Altogether, these findings suggest that the main activity against these strains may be attributed to the richness of the EOs in 1,8-cineole, but the decrease in activity could be due to the presence of a high content in spathulenol. This finding was supported by previous studies [78, 79], which reported that *1,8*-cineole had strong antibacterial activity against many important pathogens, *such as E. coli*, *S. aureus*, and *B. Subtilis*. *E. cladocalyx* was placed in the same subgroup as the previous *Eucalyptus* species in the antibacterial HCA and PCA analyses, but it was classified into another subgroup within the chemical HCA and PCA analyses, suggesting that other chemical components characterizing the oil could be involved in the total activity, such as globulol and methyl amyl acetate. Furthermore, the synergetic effect with *1,8*-cineole could produce a similar effect observed with oils rich in 1,8-cineole and poor in spathulenol. Hendry et al. (2009) and Miguel et al. (2018) [80, 81], reported that 1,8-cineole combined with other terpenes such as camphene, *α*-pinene, globulol and limonene was, by synergetic effect, more efficient against *S. aureus*, methicillin-resistant *S. aureus* (MRSA), *E. coli* and *P. aeruginosa*.

*E. lesouefii* EO, characterized by its high levels of *β*-pinene, terpinen-4-ol and sapthulenol, exhibited the best inhibition activity against both *S. aureus* and *P. aeruginosa.* However, it remains less important than other EOs, particularly against *K. pneumoniae* and *E. coli*. Comparing the variability of *S. aureus* sensivity to the oils having less concentration of the previous first three compounds and an equal or superior content of *p*-cymene, *trans*-pinocarveol, *α*-terpineol and citronellal, we could conclude that by antagonism effect, the latter compounds may be responsible for the decrease in activity. However, the increasing level of EOs' effect on the same strain could be due to a synergetic effect between *β*-pinene, terpinen-4-ol, spathulenol and other minor compounds such as aromadendrene and epiglobulol. Hammer et al. (2003) and Inouye et al. (2001) [82, 83], reported that the monoterpene alcohol terpinen-4-ol has strong antifungal and antibacterial activity, especially against *S. aureus*. However, many studies have reported that minor compounds may have synergetic or additive [84]. The correlation between the chemical composition and the antibacterial activity of the tested oils also showed that the low activity against *P. aeruginosa,* which was observed with *E. lesouefii* and *E. robusta* oils, could be due to a synergetic effect mainly between terpinen-4-ol, *β*-pinene, citronellal, *α*-terpineol and other compounds such as spathulenol, rosifoliol, *endo*-borneol, but the presence of high levels of *p*-cymene, *1,8*-cineole and the presence of other minor components such as aromadendrene, viridiflorol, globulol may considerably reduce the effect of the EO. *E. accedens* EO*,* characterized by the highest mean percentage of *α*-pinene and sharing almost the same mean percentage of *p*-cymene and *1,8*-cineole with *E. robusta* EO*,* was relatively more effective against *H. parainfluenzae.* The EOs extracted from E. melliodora and *E. bosistoana*, on the other hand, were ineffective against *H. parainfluenzae* and *H. influenzae* due to their high content of *1,8*-cineole and low content of *p*-cymene and *α*-pinene. Similarly, *E. cladocalyx* EO, which has a nearly identical content of *1,8*-cineole as *E. accedens* EO and a very low content of *α* -pinene, *p*-cymene, aromadendrene, globulol, viridiflorol, and methyl amyl acetate, did not show antibacterial activity against the two strains mentioned above.Altogether, *α*-pinene could be the principal compound responsible for the activity against *H. parainfluenzae*, whereas *p*-cymene and *α*-pinene synergically have an effect on the inhibition of growth of the two *Haemophilus* strains; *1,8*-cineole, aromadendrene, globulol, viridiflorol and methyl amyl acetate could exhibit an antagonism effect causing a significant diminution of the EO activity. This result was confirmed by the correlation analysis of the chemical composition and the antibacterial activity of *E. wandoo* EO, showing that the activity of the EOs was significantly reduced due to an antagonism effect of *1,8*-cineole and other minor compounds, such as aromadendrene, epiglobulol and viridiflorol. The comparative study of our results with those obtained by Sartorelli et al. (2007) [57], showed that the EO of *E. robusta* from Brazil, which was a chemotype of *α*-pinene (73.0%), limonene (8.3%) and *β*-pinene (6.8%), exhibited lower inhibition zone diameters (8.5, 6.3 mm) against *E. coli* and *P. aeruginosa*; respectively. However, the EOs from Congo, which were richer in *p*-cymene (27.3%), myrtenal (12.8%), and *β*-pinene (6.3%) and much poorer in 1,8-cineole (3.5%) exhibited a higher effect against *S. aureus* (22, 25 mm, izd) and *P. aeruginosa* (9, 16 mm) and a lower activity against *E. coli* (13, 15 mm, izd) [54] than those of the Tunisian *E. robusta* oil, which was richer in 1,8-cineole, *endo*-borneol citronellol and rosifolilol. This allowed us to deduce that the latter three components, which were absent in the samples from Congo, might be responsible for the high activity against *E. coli*. The oil of E. cladocalyx from Tunisia (Zerniza arboretum), which was also higher in *p*-cymene (24.72.0%), borneol (24.74.0%), and *α*-terpineol (18.84.4) than that from Mjez Elbab arboreta, had lower activity against E. coli (9.00.0 mm, izd) [38]. However, both of them were inactive against *S. aureus* and *P. aeruginosa*. The difference in activity could be due to the richness of the oils obtained from Mjez Elbab arboretum of *1,8*-cineole, globulol, viridiflorol and methyl amyl acetate. It has been reported that most terpenoids have high antimicrobial activity, and that this activity is linked to their hydroxyl group and the presence of delocalised electrons [85].

The MIC results obtained for *E. cladocalyx* against *H. influenzae* were in contradiction to the results obtained by the diffusion disc method. This difference could be related to the low diffusion ability of the EO, which in itself is highly dependent on water solubility and the ability of active components to diffuse through the agar [77, 81].

In the present study, we used two methods for antibacterial activity: the disc diffusion method and the microbroth dilution method. Each of these methods has its associated advantages and disadvantages. For the disc diffusion method, the interaction between extracts/bacteria is visually read. However, the inhibition zone could be populated with a minor subpopulation of bacteria, not detected visually; exhibiting increased antibiotic resistance, thus allowing them to grow closer to the disc. Although the disc diffusion test is relatively easy to setup and inexpensive, it does not provide quantitative data. For quantitative data, tests like the microbroth dilution method are available. Therefore, the antibacterial activity procedures depend on the method used as well as the chemical composition of tested compounds [44, 86, 87], as well as the used bacterial strains[87]. Consequently, results obtained by the disc diffusion and broth dilution methods may show a weak positive correlation or even negative correlation for some natural compounds [88].The effect of many factors on the antibacterial activity response, such as water solubility, diffusion index of the natural compound through the agar medium, and the loss of some molecules by vaporisation mainly for essential oils was reported [77, 86]. It was also known that in the case of Gram negative bacteria, the activity was also dependent on the volume and the polarity of the natural components as well as the polarity of bacteria lipopolysaccharide (LPS) layer [89]. In the present study, a difference in results was shown in the antibacterial activity of some compounds. Among them, the essential oils of *E. melliodora* and *E. bosistoana are* characterized by their high content of 1,8-cineole, known by its low hydrogen-bonding capacity [77, 90]. Therefore, their antibacterial activity against *K. pneumoniae* using the broth microdilution method, which depends on the interaction of compound molecules in solution, showed high MIC values. Additionally, discordant results were shown for *E. robusta*, *E. melliodora* and *E. wandoo* using both discussed methods against *E. coli*. Although the essential oils of these species had nearly the same inhibition zone diameter as Gentamicine®, their MIC values were not the same. Aside from the previously mentioned high content of 1,8-cineole, these three species also had a high content of monoterpene hydrocarbons (*α*-pinene and *p*-cymene), which are known for their low hydrogen-bonding capacity [77]. Altogether, we could confirm that the antibacterial activities by these two methods were not parallel [88]. Indeed, it is more reliable to use the two methods for screening the antimicrobial activity of natural compounds.

Finally, in light of the problems associated with antibiotics, i.e. bacterial resistance, EOs extracted from *E. bosistoana*, *E. robusta*, and *E. melliodora*, could be used as an alternative to treat ear infections.

## Conclusion

The chemical PCA and HCA analyses separated the EOs extracted from eight *Eucalyptus* species into seven groups. Each group constituted a chemotype. On the other hand, PCA and HCA analyses of their antibacterial activity separated them into five subgroups of *Eucalyptus* species EOs, identified by their levels of antibacterial growth inhibition. *E. melliodora and E. bosistoana* of the subgroup D_4_ were the richest species in 1,8-cineole while the highest mean percentage of *α*-pinene and *p*-cymene were detected in *E. accedens* (Subgroup D_3_) and *E. wandoo* (subgroup D_1_), respectively. The antibacterial activity of the tested *Eucalyptus* oils varied significantly between species and strains. Compared to the antibiotic Gentamicine®, *P. aeruginosa*, *H. influenzae*, *H. parainfluenzae,* and *S. aureus* were more resistant to all the tested oils. *E. robusta* and *E melliodora* oils, belonging to different chemotypes, exhibited the best inhibition zone diameter against *E. coli* and *K. pneumoniae,* respectively. In general, the highest antibacterial activity was not dependent only on a high mean percentage of one major compound such as 1,8-cineole, but also on the presence of moderate and minor compounds such as citronellal, *endo*-borneol, *α*-terpineol and rosifoliol. *E. melliodora* and *E. bosistoana* oils may have an interesting prospect in therapeutic application of some bacterial strains such as *E. coli* and *K. pneumoniae*, responsible for ear infection.

## Supplementary Information

**Additional file 1.**

## Data Availability

Data and materials are available from authors on reasonable request.

## Abbreviations

GC: Gaz chromatography
GC/MS: Gaz chromatography coupled to the mass spectroscopy
HP: Hewlett-Packard
FID: Flame ionization detector
cap: Capillary
anal: Analytical
i.d.: Internal diamaeter
EOs: Essential oils
MH: Mueller–Hinton
MIC: Minimal inhibition concentration
MBC: Minimum bactericidal concentration
IZD: Inhibition zone diameter
DMSO: Dimethyl sulfoxide
PCA: Principal components analysis
HCA: Hierarchical clusters analysis
RI: Retention index
