# Correction to: Chemical composition of essential oils of eight Tunisian *Eucalyptus* species and their antibacterial activity against strains responsible for otitis

**DOI:** 10.1186/s12906-021-03412-0

**Published:** 2021-09-28

**Authors:** Elaissi Ameur, Moumni Sarra, Derbali Yosra, Khouja Mariem, Abid Nabil, Frederic Lynen, Khouja Mohamed Larbi

**Affiliations:** 1 Chemical, Pharmacological and Gallenic Development Laboratory, University of Monastir, Faculty of Pharmacy, Avenue Avicennne, 5000 Monastir, Tunisia; 2 University of Carthage, The National Research Institute of Rural Engineering, Water and Forestry, INRGREF, Laboratory of Management and Valorization of Forest Resources, BP 10, 2080 Ariana, Tunisia; 3 Laboratory of Transmissible Diseases and Biological Active Substances LR99ES27, Faculty of Pharmacy, University of Monastir, Monastir, Tunisia; 4 High Institute of Biotechnology of Sidi Thabet, University of Manouba, Manouba, Tunisia; 5 Separation Science Group, Department of Organic and Macromolecular Chemistry, Faculty of Sciences, Ghent University, Krijgslaan 281-S4 Bis, B-9000 Ghent, Belgium

**Correction to: BMC Complement Med Ther 21, 209 (2021)**

**https://doi.org/10.1186/s12906-021-03379-y**

Following publication of the original article [1], the authors identified an error in the author name of Khouja Mariem.

The incorrect author name is: Kouja Mariem

The correct author name is: Khouja Mariem

The original article has been corrected.
