# Supplementary material for: Chemical composition of essential oils of eight Tunisian Eucalyptus species and their antibacterial activity against strains responsible for otitis
Source: BMC Complement Med Ther. 2021 Aug 12;21:209. doi: 10.1186/s12906-021-03379-y (PMC8359536; doi:10.1186/s12906-021-03379-y)
Supplement: Supplementary file 1 — Additional file 1. [file 12906_2021_3379_MOESM1_ESM.doc]

Table2 Suppl.

Chemical Composition of the Essential Oils Extracted from Leafs of Eight *Eucalyptus* Species

| Compound class and name | RI^a)^ | Content [%] | | | | | | | |
| --- | --- | --- | --- | --- | --- | --- | --- | --- | --- |
|  |  | *E. accedens* | *E. bosistoana* | *E. cladocalyx* | *E. lesoufeii* | *E. melliodora* | *E. punctata* | *E. robusta* | *E. wandoo* |
| ***Monoterpene hydrocarbons*** | | **51.2** | **16.0** | **4.6** | **33.7** | **10.6** | **40.5** | **30.1** | **47.2** |
| *α*-Thuyene | 925 | 0.6 | tr^b)^ | tr | 0.7 | tr | tr | tr | tr |
| *α*-Pinene | 932 | 38.2 | 10.8 | 3.9 | 12.8 | 9.2 | 4.2 | 15.1 | 6.5 |
| camphene | 952 | 0.1 | 0.1 | tr | 0.1 | 0.2 | 0.3 | 1.8 | 0.2 |
| Verbenene | 958 | tr | - ^c)^ | - | tr | tr | 0.1 | tr | tr |
| Thuja-2,4(10)-diene | 961 | - | - | - | - | tr | - | - | - |
| Sabinene | 972 | tr | tr | tr | 0.1 | - | tr | tr | - |
| *β*-Pinene | 976 | - | 0.3 | tr | 10.9 | 0.1 | 5.4 | 0.1 | 0.1 |
| Myrcene | 992 | 0.4 | 0.1 | tr | 0.6 | 0.1 | 0.1 | 0.1 | tr |
| δ-2-Carene | 999 | tr | tr | tr | tr | - | tr | tr | tr |
| *α*-Phellandrene | 1 005 | 2.2 | 0.2 | tr | 0.1 | 0.2 | 0.2 | 0.2 | tr |
| *α*-Terpinene | 1 016 | 0.1 | tr | tr | 0.1 | tr | 0.1 | 0.1 | tr |
| *p*-Cymene | 1 024 | 8.6 | 4.0 | 0.4 | 7.7 | 0.4 | 28.7 | 11.8 | 35.8 |
| Limonene | 1 028 | 0.7 | 0.3 | - | - | 0.3 | 0.6 | - | 0.4 |
| *cis-β*-Ocimene | 1 037 | - | - | 0.1 | tr | - | 0.1 | - | tr |
| *trans*-β-Ocimene | 1 048 | tr | - | - | tr | - | 0.1 | tr | - |
| *γ*-Terpinene | 1 057 | 0.1 | 0.1 | 0.1 | 0.3 | tr | 0.1 | 0.3 | 3.9 |
| *p*-Mentha-3,8-diene | 1 065 | - | - | - | tr | - | - | tr | - |
| Terpinolene | 1 089 | 0.2 | 0.1 | 0.1 | 0.2 | 0.1 | 0.5 | 0.6 | 0.2 |
| ***Monoterpene oxides*** | | **28.1** | **52.8** | **39.2** | **38.1** | **66.3** | **21.5** | **27.4** | **37.8** |
| 1,8-Cineole | 1 030 | 28.1 | 52.7 | 39.2 | 38.0 | 66.3 | 20.7 | 26.5 | 37.7 |
| *trans*-Linaloloxide | 1 069 | tr | tr | - | tr | tr | 0.1 | 0.4 | - |
| *α*-Pineneoxide | 1 100 | tr | tr | - | 0.1 | - | 0.1 | - | tr |
| *cis*-Rose oxide | 1 108 | - | tr | tr | - | - | - | - | - |
| *trans*-Rose oxide | 1 121 | tr | - | tr | - | - | 0.6 | 0.5 | tr |
| ***Monoterpene ketones*** | | **0.7** | **1.6** | **0.6** | **1.7** | **1.4** | **12.2** | **0.9** | **0.9** |
| Fenchone | 1 083 | - | - | tr | tr | - | 0.1 | - | - |
| 2-Nonanone | 1 094 | - | - | - | tr | tr | - | - | - |
| Nopinone | 1 125 | tr | 0.1 | tr | 0.2 | 0.1 | 0.3 | tr | tr |
| camphor | 1 148 | tr | tr | tr | 0.1 | tr | 0.4 | - | tr |
| Pinocarvone | 1 166 | 0.2 | 0.2 | - | 0.1 | 1.0 | 0.7 | - | 0.3 |
| Cryptone | 1 186 | 0.1 | 0.6 | 0.3 | 0.8 | - | 8.4 | - | 0.2 |
| Carvotanacetone | 1 219 | - | - | - | tr | - | - | - | - |
| Verbenone | 1 227 | 0.2 | 0.5 | 0.1 | 0.3 | - | 1.5 | 0.6 | 0.2 |
| Carvone | 1 244 | tr | 0.1 | tr | 0.1 | 0.1 | 0.3 | tr | tr |
| Carvotonacetone | 1 249 | tr | 0.1 | tr | - | 0.1 | 0.2 | 0.1 | tr |
| Piperitone | 1 254 | - | tr | tr | 0.2 | - | 0.3 | 0.1 | tr |
| Geranylacetone | 1 454 | 0.1 | - | tr | - | 0.1 | tr | 0.1 | tr |
| ***Monoterpene aldehydes*** | | **0.2** | **0.4** | **0.5** | **1.4** | **0.1** | **4.0** | **4.0** | **0.1** |
| Bergamal | 1 052 | tr | tr | tr | tr | - | tr | tr | - |
| citronellal | 1 157 | 0.1 | 0.1 | 0.4 | 1.0 | 0.1 | 0.2 | 3.5 | tr |
| Myrtenal | 1 202 | 0.1 | 0.2 | tr | 0.1 | tr | 0.3 | - | - |
| Neral | 1 237 | - | 0.1 | - | - | - | - | 0.1 | - |
| Cuminaldehyde | 1 239 | tr | tr | tr | 0.1 | tr | 2.1 | 0.2 | tr |
| *2-trans*- decenal | 1 264 | - | - | 0.1 | tr | - | 0.1 | - | - |
| Geranial | 1 265 | tr | - | - | 0.1 | tr | 0.1 | tr | - |
| Phellandral | 1 274 | - | tr | - | tr | tr | 1.1 | - | tr |
| *p*-Menth-1-en 7-al | 1 278 | - | tr | - | 0.1 | - | - | - | - |
| 3-oxo-para-Menth-1-en-7-al | 1 334 | - | tr | - | tr | - | 0.1 | tr | - |
| **Monoterpene alcohols** | | **4.0** | **5.6** | **3.2** | **11.0** | **9.2** | **9.1** | **20.3** | **5.3** |
| Linalool | 1 102 | - | tr | - | - | - | - | - | - |
| *D*-fenchyl alcohol | 1 113 | 0.1 | 0.1 | 0.1 | 0.2 | 0.4 | 0.3 | 2.2 | 0.2 |
| Exofenchol | 1 120 | tr | 0.1 | tr | 0.2 | 0.1 | - | tr | - |
| Isopinocarveol | 1 129 | - | - | - | - | tr | 0.1 | tr | - |
| *trans*-Pinocarveol | 1 138 | 2.3 | 3.1 | 2.0 | 3.2 | 4.0 | 4.2 | 5.3 | 2.2 |
| Sabinol | 1 144 | 0.1 | tr | tr | 0.2 | tr | 0.3 | 0.2 | tr |
| *endo*-Borneol | 1 162 | 0.5 | 0.9 | 0.3 | 0.4 | 0.7 | 1.1 | 6.0 | 0.4 |
| Borneol | 1 171 | tr | tr | 0.1 | 1.8 | tr | 0.3 | 0.4 | tr |
| Terpinen-4-ol | 1 176 | 0.4 | 0.2 | 0.3 | 3.3 | 0.3 | 0.4 | 0.3 | 1.1 |
| trans-p-Menth-1(7),8-dien-2ol | 1 188 | - | - | - | - | 0.9 | - | - | - |
| α-Terpineol | 1 191 | 0.5 | 0.9 | 0.2 | 1.2 | 1.6 | 0.5 | 6.7 | 1.2 |
| *p*-Cymen-8-ol | 1 196 | 0.2 | 0.1 | tr | - | 0.1 | 3.0 | 0.4 | 0.1 |
| *trans*-Piperitol | 1 206 | tr | tr | - | - | - | - | 0.2 | - |
| Myrtenol | 1 209 | tr | tr | tr | 0.3 | 0.1 | 0.7 | 0.1 | - |
| *trans*-carveol | 1 219 | 0.1 | 0.2 | 0.2 | 0.2 | 0.3 | 0.4 | 0.7 | 0.1 |
| *cis-p*-Mentha-1(7),8-dien-2-ol | 1 228 | - | - | - | - | 0.9 | - | - | - |
| *cis*-Carveol | 1 232 | - | - | tr | - | - | - | - | - |
| Geraniol | 1 258 | 0.1 | tr | - | tr | - | tr | - | tr |
| Cuminol | 1 290 | - | tr | tr | 0.1 | tr | 1.1 | 0.2 | 0.1 |
| Cymen-7-ol | 1 292 | - | tr | tr | - | tr | - | 0.3 | 0.1 |
| ***Phenols*** |  | **0.4** | **0.2** | **0.1** | **0.4** | **0.2** | **1.1** | **0.6** | **0.8** |
| *p*-Cresol | 1 071 | tr | - | tr | - | - | 0.2 | tr | tr |
| *m*-Cresol | 1 074 | - | - | tr | - | - | tr | tr | - |
| Thymol | 1 298 | tr | 0.1 | tr | 0.3 | tr | 0.3 | 0.5 | 0.3 |
| Carvacrol | 1 302 | 0.3 | 0.1 | - | 0.1 | 0.1 | 0.6 | tr | 0.5 |
| Eugenol | 1 357 | 0.1 | - | - | tr | 0.1 | - | - | - |
| ***Monoterpene esters*** | | **0.1** | **0.3** | **0.3** | **0.6** | **1.1** | **0.3** | **0.6** | **0.5** |
| Isoamyl-2-methyl butyrate | 1 097 | - | - | 0.1 | tr | tr | - | 0.1 | - |
| Isoamyl isovalerate | 1 106 | tr | 0.1 | 0.1 | tr | 0.6 | 0.2 | 0.1 | tr |
| Fenchyl acetate | 1 230 | tr | 0.1 | 0.1 | 0.1 | 0.3 | 0.1 | 0.3 | 0.1 |
| Isobornyl acetatae | 1 287 | - | tr | - | 0.4 | 0.1 | - | 0.1 | 0.4 |
| Methyl geranate | 1 325 | - | - | - | tr | - | - | tr | tr |
| Myrtenyl acetate | 1 329 | - | - | - | 0.1 | tr | tr | - | - |
| Citronellyl acetate | 1 354 | - | - | tr | - | - | - | - | - |
| ***Methyl ether phenol*** | | **-** | **0.1** | **-** | **-** | **tr** | **-** | **tr** | **tr** |
| Methyl eugenol | 1 401 | - | 0.1 | - | - | tr | - | tr | tr |
| ***Sesquiterpene hydrocarbons*** | | **2.0** | **10.5** | **14.4** | **1.1** | **5.3** | **0.4** | **1.3** | **0.4** |
| Bicycloelemene | 1 338 | - | - | 0.1 | tr | tr | tr | tr | - |
| *α*-Cubebene | 1 346 | 0.1 | - | 0.1 | - | 1.7 | - | - | - |
| *α*-Copaene | 1 372 | - | tr | tr | 0.1 | tr | - | 0.1 | - |
| Isoledene | 1 375 | - | tr | 0.1 | - | - | - | - | - |
| *α*-Elemene | 1 377 | - | - | - | - | 0.1 | - | - | - |
| *β*-Elemene | 1 392 | tr | tr | 0.1 | - | - | - | tr | - |
| *α*-Gurjunene | 1 404 | tr | 0.1 | 0.3 | tr | 0.1 | - | 0.1 | - |
| *trans*-Caryophyllene | 1 418 | 0.7 | tr | tr | - | 0.5 | tr | - | 0.1 |
| *β*-Gurjunene | 1 431 | - | 0.3 | 0.3 | tr | tr | - | 0.3 | tr |
| Aromadendrene | 1 438 | 0.2 | 7.3 | 8.7 | 0.4 | 0.6 | 0.1 | 0.2 | 0.1 |
| *α*-Humulene | 1 448 | - | - | - | tr | - | - | tr | - |
| Alloaromadendrene | 1 460 | 0.1 | 1.5 | 1.3 | 0.4 | 0.3 | 0.1 | 0.1 | tr |
| *γ*-Gurjunene | 1 472 | - | 0.1 | 0.2 | tr | tr | - | tr | tr |
| *trans*-Cadina-1(6),4-diene | 1 477 | - | 0.2 | tr | - | - | - | - | - |
| *trans-cis-α*-Farnezene | 1 485 | - | 0.2 | 0.6 | - | 0.1 | - | 0.2 | - |
| *α*-amorphene | 1 486 | - | - | tr | - | - | - | - | 0.1 |
| *β*-Selinene | 1 489 | 0.1 | - | - | - | - | - | - | - |
| Ledene | 1 492 | tr | tr | 1.6 | tr | - | 0.1 | tr | tr |
| *α*-Muurolene | 1 493 | - | - | - | - | - | - | - | - |
| Bicyclogermacrene | 1 494 | 0.6 | 0.1 | tr | tr | 0.8 | - | tr | - |
| (*Z-Z) -α*-Farnesene | 1 496 | - | 0.1 | 0.1 | tr | - | tr | - | tr |
| Cis- α -bisabolene | 1 496 | - | - | - | - | - | - | - | - |
| δ-Guaiene | 1 500 | - | - | tr | tr | 0.1 | - | - | - |
| Germacrene | 1 506 | tr | - | 0.1 | tr | tr | - | 0.1 | - |
| δ-Cadinene | 1 523 | 0.1 | tr | tr | tr | - | - | tr | tr |
| *trans*-Calmenene | 1 529 | - | tr | tr | tr | tr | - | tr | - |
| *trans*-Cadina-1,4-Diene | 1 535 | - | - | tr | - | tr | - | - | - |
| Germacrene | 1 559 | 0.1 | 0.6 | 1.0 | 0.1 | 0.2 | tr | 0.1 | tr |
| *β*-Selinene | 1 589 | - | - | - | - | 0.7 | - | - | - |
| ***Sesquiterpene alcohols*** | | **8.9** | **9.7** | **21.6** | **7.5** | **2.9** | **2.4** | **8.1** | **2.8** |
| Elemol | 1 543 | tr | tr | 0.2 | tr | - | - | tr | - |
| epiglobulol | 1 552 | tr | 0.9 | 2.3 | 0.1 | 0.1 | 0.1 | 0.3 | tr |
| Palustrol | 1 562 | - | tr | tr | - | - | - | tr | - |
| Ledol | 1 567 | 0.2 | 0.4 | tr | - | 0.2 | tr | 0.4 | tr |
| Spathulenol | 1 577 | 2.5 | 4.1 | 0.2 | 4.6 | 0.5 | 1.6 | 0.2 | - |
| Globulol | 1 584 | 2.4 | 2.3 | 12.7 | 0.8 | 1.2 | - | 1.4 | 0.1 |
| Viridiflorol | 1 591 | 1.0 | 0.7 | 2.6 | 0.1 | 0.2 | 0.1 | 0.3 | 0.2 |
| Guaiol | 1 603 | 0.3 | - | - | - | tr | 0.1 | - | - |
| atlantol | 1 610 | - | 0.2 | 0.8 | tr | - | - | - | tr |
| Rosifoliol | 1 612 | 0.2 | 0.4 | 1.7 | tr | tr | 0.1 | 5.2 | tr |
| Isospathulenol | 1 633 | 0.3 | 0.1 | 0.3 | 0.3 | 0.2 | tr | 0.2 | 0.7 |
| Hinesol | 1 642 | 0.6 | 0.1 | 0.1 | - | 0.1 | - | 0.1 | 1.2 |
| *β*-Eudesmol | 1 645 | 0.7 | 0.2 | 0.5 | 1.1 | - | tr | 0.1 | 0.3 |
| α-Eudesmol | 1 647 | 0.8 | 0.1 | 0.2 | 0.4 | 0.2 | 0.1 | tr | - |
| *7-epi-α*-Eudesmol | 1 659 | - | tr | tr | - | - | 0.1 | - | 0.1 |
| *trans-cis*-farnesol | 1 732 | - | tr | 0.1 | - | tr | - | - | - |
| ***Sesquiterpene oxides*** | | **-** | **-** | **-** | **-** | **-** | **1.5** | **-** | **-** |
| Caryophylleneoxide | 1 583 | - | - | - | - | - | 1.5 | - | - |
| ***Sesquiterpene aldehydes*** | | **-** | **0.1** | **tr** | **-** | **-** | **-** | **-** | **-** |
| isobicyclogermacrenal | 1 734 | - | 0.1 | tr | - | - | - | - | - |
| ***Oxygenated aromatics*** | | **tr** | **tr** | **tr** | **-** | **tr** | **tr** | **tr** | **tr** |
| Benzaldehyde | 966 | tr | tr | tr | - | tr | tr | tr | tr |
| **Ketones** |  | **-** | **tr** | **tr** | **-** | **-** | **tr** | **tr** | **tr** |
| 6-Methyl-5- Hepten-2-one | 987 | - | tr | tr | - | - | tr | tr | tr |
| ***Aliphatic ester*** | | **tr** | **tr** | **8.9** | **tr** | **tr** | **tr** | **tr** | **tr** |
| Methylamylacetate- | 900 | tr | tr | 8.9 | tr | tr | tr | tr | tr |
| **Total identified** | | **95.8** | **97.3** | **93.6** | **95.6** | **97.7** | **96.4** | **96.1** | **95.9** |

^a)^RI : Retention index determined on HP5 cap. Column; ^b)^ tr: Trace (<0.1); ^c)^ : Not detected.
